# Wastage in the British Army

**Published:** 1918-09

**Authors:** 


### WASTAGE OF MEN IN WAR

**Wastage in the British Army.** Lieutenant-General BURTCHAEL, D. M. S., R. A. M. C. The speaker, after a word of hearty appreciation of the services of American medical officers with the British forces, which is printed among the editorial comments, spoke as follows:

Wastage in an Army may be looked at from a great many points of view — the net wastage or total loss may be taken in relation to either the entire strength in the field or in relation to the front line units alone. Formerly we considered wastage only as affecting the fighting troops. Now the number of men employed on labor behind the fighting line has assumed such large proportions it is necessary to watch wastage in that element as well as amongst fighting men, and to have regard to the fact that a fairly large proportion of the men who drop out as non-effectives from the fighting ranks go towards forming efficient units in labor groups.

Ordinarily wastage or loss of fighting strength is caused in one of two ways, that is to say, by casualties in battle, or through sickness. The proportion of wounded to sick in the British Army last year in every thousand men admitted to hospital was 374 wounded to 626 non-wounded or "sick".

Primary wastage from wounds cannot be controlled by the Medical Service, but it is possible to influence the net loss that might occur from wounds by adopting certain procedures and lines of treatment with a view to getting men back to duty in the shortest possible time unless their injuries are such as to cause permanent incapacity for military service. In looking at the tabular statements of the disposal of sick and wounded you must remember that the term "sick", as here used in contra-distinction to "wounded", is really a misnomer as under that heading are included all forms of injuries other than those caused by weapons in action as well as disabilities which would usually be classified as surgical rather than medical. Also no great epidemic influenced the figures. Fortunately there has been no serious outbreak of epidemic infectious disease in the British Armies and we have not had to concern ourselves with wastage from that cause. However, in an Army one must be constantly on the look-out for the possibility of extension into epidemic form of the principal infectious diseases, nearly all of which are permanently represented by a few cases, and we have to guard against the introduction and spread of the germs of diseases not usually present. Infectious disease, if uncontrolled, might be the cause of a tremendous wastage. As a matter of fact the only infectious disease of any consequence from the point of view of temporary loss of strength that we have experienced was the recent epidemic of influenza which for about a week assumed serious proportions. It spread rapidly and, although men were only off duty from 4 to 7 days, the numbers affected were for the moment serious in relation to the military situation. Fortunately it passed off quickly.

The total wastage in the British Armies in France is represented by deaths and evacuation to the United Kingdom — and the numbers lost have to be replaced by reinforcements. As a matter of convenience these numbers are watched in their relation to the disposal of all cases that come under treatment in the Casualty Clearing Stations and Base Hospitals combined, and not directly in relation to the total strength of troops — but I may say that the daily wastage per 1000 of strength is small and generally below the figure usually taken as normal. On the above basis the rates per 1000 of hospital cases are :

| | All cases. | Sick. | Wounded. |
|---|---|---|---|
| Discharged to duty. . . . . . . | 413 | 521 | 232 |
| To Medical Board. . . . . . . . | 56 | 82 | 18 |
| Evacuated . . . . . . . . . . . | 502 | 391 | 682 |
| Died. . . . . . . . . . . . . . | 29 | 6 | 68 |
| Totals . . . . . | 1000 | 1000 | 1000 |

Factors other than the actual physical condition often influence the numbers transferred to England. For instance, temporary pressure on the bed accommodation in times of battle sometimes results in cases being evacuated which would be retained in France and come up for disposal locally in normal circumstances. Medical Board cases are those of a doubtful nature which are seen for assessment by Standing Medical Boards as to the category of physical fitness in which they should be placed. About 20 per cent. of all such cases eventually become category " A " or fit for General Service; otherwise they pass into the " B " class and are allotted to employments other than active combatant duties.

All casualties whether through wounds or sickness occurring in an Army established in the front line must be replaced from the base by reinforcements which are held in readiness at various Depots. These reinforcements on arrival in France are inspected for infectious diseases and for various disabilities of a general nature which may cause rejection for service at the front.

If we had to deal only with a normal man, free of any defect, he should never become a casualty from sickness unless he is brought into contact with infection, given infected food or drink, or in the course of time falls a victim to disease due directly to rigorous weather or other conditions incidental to front line life. But as a matter of fact, few men are absolutely normal and no one knows how many men out of a given number are really normal. Many have slight defects which existed on enlistment and which may easily be aggravated by exposure and cause incapacity to carry on. This class produces the greater part of the so called "sick". Only one or two conditions which we know to be preventable, such as trench fever and P. U. O., give a high percentage in the total sick wastage. Thanks to the work of the Commission appointed by the American Red Cross to collaborate with the British Committee, we now know the cause of trench fever and it may be possible to completely eradicate it as a source of wastage.

In the front or battle line the soldier comes under the care and supervision of the regimental medical officer, and whether he falls out to swell the sick wastage figure of the unit or not depends to a very great extent upon the care, experience, personality, and knowledge of men as well as of medicine which this officer brings to bear on his duties. The proportion of wastage varies in the front line directly with the aptitude of the unit medical officer. Some medical officers are able to keep the men fit and cheerful and in the line by taking an interest in each individual by constant inspections and by making light of the ailments which are of little consequence.

On the other hand those medical officers who do not get in touch with their men and do not carry out the customary inspections of a sanitary or hygienic nature are soon surrounded by men who readily grow sick. At all times there are certain men in front line units who feel weary of war or a bit jaded and tired, or who have genuinely enough a pain here or there. The officer who is not in touch with his men often takes some quite insignificant symptom to be an indication of real sickness and passes the soldier back for observation. This practice results in men being constantly passed back who would have readily carried on their work if the medical officer had taken the line of cheerful encouragement. A regimental medical officer has a splendid opportunity of achieving success by endeavouring to produce a good psychological effect upon his men.

It is very interesting to investigate the problems which lie behind the factors which cause a man to "go sick" or not. One of your most distinguished surgeons brought before us some time ago a scheme for investigating the psychology of the soldier in the various circumstances in which he may be placed — when in the trenches, when going over the top and even at the moment when he is plunging a bayonet into the stomach of an enemy. Unfortunately the practical difficulties in the way were great and the scheme has not yet been put into operation.

As regards the proportion of men who on passing out of hospitals and convalescent depots are sent to the Medical Board depots for physical classification, the disabilities per 1000 of all cases which give the highest ratio of entries into the " B " class are age and rheumatism 150, various conditions returned under the heading V. D. H. or D. A. H. 120, debility and anemia 100, old wounds or injuries 70, defects of vision 70 and myalgia 40. These terms are often wrongly applied and very often they do not represent correctly the actual disease or true disability from which a man may be suffering, but that is unavoidable when dealing with very large numbers of men often passed rapidly from the front to the base at times when there is no time or indeed necessity to enter a more exact diagnosis. Some of the conditions just mentioned, such as doubtful rheumatism, minor irregularities of the heart, debility, slight injuries or chronic affection of the joints, would ordinarily justify us in eliminating men at once as not fit to be sent back to the front at all. But that is not always a good policy. In many instances it is best to make little of a man's disability and pass him into the stream of reinforcements. Possibly any doubtful man may come back, but a large number of semi-defectives sent out as " A " do stick at the front and do not come back. Men unable to carry on, on account of such disabilities as hernia, piles, and deformities, are not sent back to the base through hospitals, but are passed down from the front direct to the base depot and from there they are brought before the Medical Board. If the Board does not agree with the judgment of the divisional medical officers, the medical inspector of drafts is informed and he, acting as a liaison officer with the division and comparing the conditions of the man assumed to have been wrongly assessed at the front with those of other men reviewed, endeavors to bring about an understanding as to the types of disability and physical conditions of a defective nature which justify a man's being retained at the front for duty.

Convalescent Depots give a very large yield of fit men — some 800 out of every thousand passed into the depots from hospitals. There are many important points concerning the control and treatment of men in these convalescent depots. The object is to take men out of the hospitals and put them through an institution run on military lines, where they are medically supervised, but otherwise treated as soldiers and not as sick or non-effective men. Measures are used to get them into fit condition both physically and mentally and to keep up a military atmosphere. They attend parades and are given physical exercises, route marches, etc., but each exercise is of short duration. They get good food and plenty of amusement — a band is an essential. But above all, young active medical officers, if possible those who have had experience in the front lines, are placed in command. It is their duty to get into personal touch with the men so far as possible, to join in their sports, march out with them, and at the same time closely observe and assess the condition of each man. When men stay too long in hospital they frequently become depressed, hospitalized, and temporarily degenerate mentally. Some persons, especially sympathetic nurses, are inclined to keep patients too long in the hospital. This is not good for the individual man or his unit. Perhaps too few of those who directly or indirectly influence the stay of a man in hospital, consider that every day he is unnecessarily detained there some one else must do his work. The policy is to get every man up and about and out as soon as possible and make him feel that he is a soldier and not a sick man. To keep men cheerful and happy is half the battle and is the kindest and best of treatments for all those with temporary or more or less imaginary illnesses. A man's mental outlook should be put right before he is passed out of hospital or out of medical charge.

I have just given you an outline of the system through which men circulate from the fighting ranks to hospitals and back again—some of the officers who control various points in that system will tell you something of the details which I have not touched upon.

